# ChatGPT-4–Driven Liver Ultrasound Radiomics Analysis: Diagnostic Value and Drawbacks in a Comparative Study

**DOI:** 10.2196/68144

**Published:** 2025-06-30

**Authors:** Laith R Sultan, Shyam Sunder B Venkatakrishna, Sudha A Anupindi, Savvas Andronikou, Michael R Acord, Hansel J Otero, Kassa Darge, Chandra M Sehgal, John H Holmes

**Affiliations:** 1 Department of Radiology Children's Hospital of Philadelphia Philadelphia, PA United States; 2 Department of Radiology University of Pennsylvania Philadelphia United States; 3 Department of Biostatistics, Epidemiology and Informatics University of Pennsylvania Philadelphia, PA United States

**Keywords:** ChatGPT-4, artificial intelligence, large language models, radiomics, ultrasound imaging, quantitative image analysis, liver disease, radiology workflow

## Abstract

**Background:**

Artificial intelligence (AI) is transforming medical imaging, with large language models such as ChatGPT-4 emerging as potential tools for automated image interpretation. While AI-driven radiomics has shown promise in diagnostic imaging, the efficacy of ChatGPT-4 in liver ultrasound analysis remains largely unexamined.

**Objective:**

This study aimed to evaluate the capability of ChatGPT-4 in liver ultrasound radiomics, specifically its ability to differentiate fibrosis, steatosis, and normal liver tissue, compared with conventional image analysis software.

**Methods:**

Seventy grayscale ultrasound images from a preclinical liver disease model, including fibrosis (n=31), fatty liver (n=18), and normal liver (n=21), were analyzed. ChatGPT-4 extracted texture features, which were compared with those obtained using interactive data language (IDL), a traditional image analysis software. One-way ANOVA was used to identify statistically significant features differentiating liver conditions, and logistic regression models were used to assess diagnostic performance.

**Results:**

ChatGPT-4 extracted 9 key textural features—echo intensity, heterogeneity, skewness, kurtosis, contrast, homogeneity, dissimilarity, angular second momentum, and entropy—all of which significantly differed across liver conditions (*P*<.05). Among individual features, echo intensity achieved the highest *F*_1_-score (0.85). When combined, ChatGPT-4 attained 76% accuracy and 83% sensitivity in classifying liver disease. Receiver operating characteristic analysis demonstrated strong discriminatory performance, with area under the curve values of 0.75 for fibrosis, 0.87 for normal liver, and 0.97 for steatosis. Compared with IDL image analysis software, ChatGPT-4 exhibited slightly lower sensitivity (0.83 vs 0.89) but showed moderate correlation (*r*=0.68, *P*<.001) with IDL-derived features. However, it significantly outperformed IDL in processing efficiency, reducing analysis time by 40%, and highlighting its potential for high throughput radiomic analysis.

**Conclusions:**

Despite slightly lower sensitivity than IDL, ChatGPT-4 demonstrated high feasibility for ultrasound radiomics, offering faster processing, high-throughput analysis, and automated multi-image evaluation. These findings support its potential integration into AI-driven imaging workflows, with further refinements needed to enhance feature reproducibility and diagnostic accuracy.

## Introduction

In recent years, advancements in artificial intelligence (AI) have transformed various fields, and one notable application is in the realm of medical imaging [1-6]. AI holds significant potential in revolutionizing the field of medical imaging, as it can automate numerous tasks and even surpass human abilities in specific areas, whether it be in diagnostic or interventional applications [7]. Integrating AI with ultrasound imaging is particularly compelling. Unlike other imaging modalities, ultrasound relies heavily on human operators [8,9]. This dependence on human expertise presents unique challenges, especially with the growing use of portable ultrasound devices. These devices are increasingly used by a diverse range of health care providers, including nonradiologists, who may have varying levels of training and experience [10]. AI algorithms offer a powerful solution to mitigate the challenges associated with operator dependency in ultrasound imaging. These algorithms can play a crucial role in the automated detection of anomalies and significant findings, providing not only descriptive analysis but also valuable diagnostic guidance [11-13]. This capability is particularly beneficial for less experienced operators or in situations where expert radiologists are not readily available in regions with limited medical resources. The integration of AI in ultrasound imaging can lead to more accurate and efficient diagnostic processes, reducing the likelihood of human error and improving patient outcomes [12-17].

ChatGPT is an advanced and powerful AI natural language processing model developed by OpenAI and was designed to comprehend and generate human-like text responses [18]. Having been extensively trained on a diverse corpus of data, ChatGPT has cultivated the capacity to grasp context, acquire knowledge from examples, and produce cohesive responses [19]. Consequently, it has evolved into a versatile tool applicable to a wide array of uses, including health care and medical imaging [20-26]. In health care, its capacity to process and interpret vast amounts of information can support medical diagnostics, patient communication, and research. The latest version, ChatGPT-4, expands its ability to multimodal interactions, including image processing and potential capabilities in audio and video formats [27-29]. This enhancement is especially beneficial in health care, where it can analyze medical imagery, assist in creating educational materials, and offer visually descriptive assistance in patient care. By integrating advanced image analysis and generation, ChatGPT-4 stands poised to transform how AI supports health care professionals, offering tools for more accurate diagnoses, treatment planning, and patient engagement through rich, interactive media.

In this study, we explore the potential of ChatGPT-4 in ultrasound imaging, particularly its capabilities in radiomics analysis for detailed tissue texture characterization. We focus on using ChatGPT-4–based radiomics to detect 3 distinct liver tissue types—normal, fibrotic, and fatty liver—using ultrasound images. To address challenges related to clinical data security, patient privacy, and ethical compliance, the liver ultrasound images in our study were sourced from an animal model. We then compared the findings generated by ChatGPT-4 with those obtained from conventional image analysis software. Our exploration highlights the potential of ChatGPT-4 to enhance research efforts and future clinical applications by improving the accuracy of quantitative image analysis.

Beyond radiomics analysis, we evaluated ChatGPT-4 as a tool for distinguishing normal from abnormal cases based on imaging findings. We aimed to demonstrate its capability as a supportive tool in clinical settings. Such a tool could significantly reduce the workload of radiologists by efficiently filtering out normal cases, allowing them to focus their expertise on more complex and abnormal cases. This expanded exploration highlights the promising role of ChatGPT-4 in enhancing diagnostic accuracy and supporting clinical decision-making in liver disease detection.

## Methods

### Image Data Acquisition

Seventy B-mode grayscale ultrasound images acquired from validated rat liver disease models [30-32] were used for analysis. The images were distributed across 3 categories of liver health: fibrosis (n=31), steatosis (fatty liver) (n=18), and normal (n=21). To maintain consistency and reliability in the analysis, the imaging parameters were standardized, including transducer frequency, gain settings, imaging depth, focus, and dynamic range. These standardizations ensured that the liver tissue’s echogenicity and overall image quality were consistent across all samples, allowing for accurate comparisons between the different health states. Additionally, each analysis focused on a single image depicting a section of the right lobe of the liver. The right lobe was chosen due to its larger size and easier accessibility, which provided a more representative and consistent area for imaging and subsequent histopathological validation. The liver pathology in these images was validated with histopathology, further ensuring the accuracy of the ultrasound-based categorization.

### Ultrasound Image Analysis by ChatGPT-4

#### Overview

**Figure 1:**
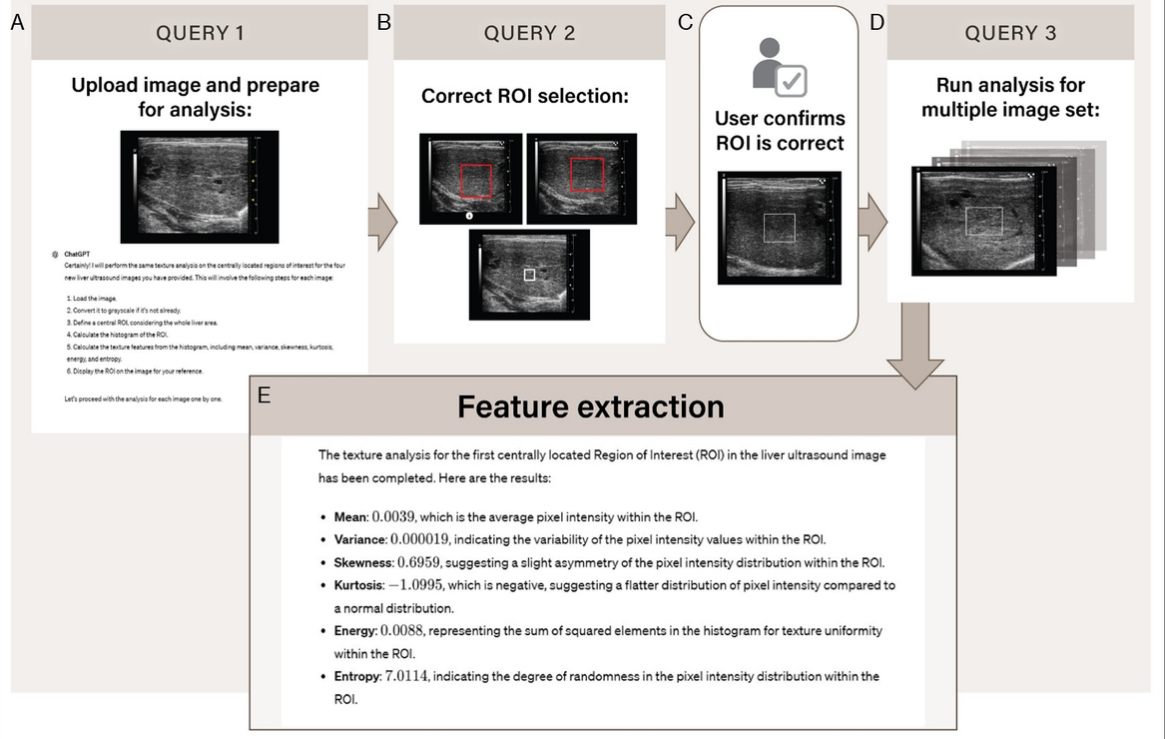
ChatGPT-4–assisted liver ultrasound image radiomics analysis workflow. The image illustrates the stepwise process of liver ultrasound texture analysis using ChatGPT-4. The process begins with uploading the image and preparation for analysis (query 1), where ChatGPT-4 performs texture analysis based on a selected ROI. In query 2, the user verifies and corrects the ROI selection. The ChatGPT-4 interface allows the user to refine the ROI to ensure accurate analysis. Once confirmed, the system proceeds to apply the same process to a series of images (query 3). Feature extraction details the analysis outputs, including texture metrics such as mean, variance, skewness, kurtosis, energy, and entropy, which provide insights into pixel intensity distribution and texture uniformity within the selected ROI. Figure prepared by Brittany Bennett, CMI. ROI: region of interest.

We leveraged the advanced capabilities of ChatGPT-4 for radiomics analysis of ultrasound images. ChatGPT-4 was used to select regions of interest (ROIs), extract radiomic features, and classify liver disease conditions. These critical steps are depicted in Figure 1.

#### Region of Interest Delineation

The first critical step involved selecting a region of interest (ROI) within the liver tissue depicted in each ultrasound image (Figures 1B and 1C). ROIs were automatically defined using ChatGPT-4’s advanced algorithms. Upon receiving the query, ChatGPT-4 initially proposed an ROI based on its automated analysis of the image, highlighting a region that it determined to be representative of the liver parenchyma. Users then refined these suggestions to ensure alignment with clinical standards, making adjustments as necessary to ensure that the selected area was optimal for analysis meticulously excluding artifacts such as vascular structures, acoustic shadows, and reverberation. This interactive process allowed for fine-tuning of the ROI, combining the computational efficiency of ChatGPT-4 with the expert judgment of the user. Once the ROIs were verified for accuracy in an analyzed image, they were replicated across 10 subsequent ultrasound images, which were then uploaded for subsequent radiomics analysis using a batch processing approach (Figure 1). Having liver images captured consistently in the same plane and region facilitated the reproducibility of ROI placement across the images. This method significantly enhanced the efficiency of our analysis, allowing for a more comprehensive assessment of liver tissue samples

#### Feature Extraction

Feature extraction was conducted in batches of 10 ultrasound images, the maximum allowed by ChatGPT-4, requiring multiple sessions to analyze all 70 cases. However, conducting analyses across different sessions introduced variability—some features were occasionally omitted, while others appeared inconsistently across sessions. To mitigate this session-dependent variability and ensure consistency in feature extraction, we implemented a standardized approach. At the beginning of each session, we carefully refined the prompts provided to ChatGPT-4 to align with previously extracted features (Table S1 in Multimedia Appendix 1). Missing features were explicitly requested, and any inconsistently appearing features were excluded. If ChatGPT-4 returned incomplete or inconsistent features, prompts were reissued or clarified until the correct output was obtained. Our final analysis included only features that were consistently and reliably extracted across all sessions. This approach minimizes session-to-session variation while maintaining reproducibility across users.

ChatGPT-4 extracted a comprehensive set of radiomic features to characterize liver tissue texture [33-35]. These included first-order statistics and second-order texture features. First-order statistics are quantitative measures such as mean intensity, variance (heterogeneity), skewness, and kurtosis, reflecting pixel intensity distribution. Second-order texture features are derived from the gray-level co-occurrence matrix (GLCM), and these features include contrast, homogeneity, entropy, and angular second momentum (ASM), providing deep insights into spatial relationships and textural heterogeneity within the ROI.

#### Machine Learning for Feature Model Assessment

The extracted radiomic features were used to develop a diagnostic model based on logistic regression, a method selected for its interpretability and clinical relevance. The model was configured with L2 regularization, and the regularization strength parameter (C) was optimized through grid search over a predefined range [36,37]. The liblinear solver was used for its suitability with small datasets, and the maximum number of iterations was set to 1000 to ensure model convergence. The dataset was divided into training (60%), testing (20%), and validation (20%) subsets using stratified random sampling to maintain a balanced representation across liver disease categories (Table S2 in Multimedia Appendix 1). Hyperparameter tuning was performed using a grid search to optimize model performance. Specifically, the regularization strength parameter (C) in the logistic regression model was adjusted to balance model fit and prevent overfitting [37]. A range of C values (eg, 0.001 to 100) was evaluated, and the optimal configuration was selected based on performance on the test set. To maintain methodological rigor, the test set was used exclusively during hyperparameter tuning, while the validation set was reserved for final model evaluation. The 3-way split ensured an unbiased assessment of model generalizability. Key metrics, including accuracy, sensitivity, specificity, and the area under the receiver operating characteristic (ROC) curve (AUC), were used to quantify diagnostic precision.

#### Ultrasound Image Analysis by Interactive Data Language–Based Software

Concurrently, the same ultrasound images were analyzed using an established interactive data language (IDL)–based tool designed for image analysis [33,38]. For this analysis, ROIs within the liver were manually defined using a specialized tool, which ensured the precise selection of the target areas based on the same selection criteria mentioned earlier. ROI delineation was performed manually by expert users, ensuring the precise inclusion of clinically relevant areas and the exclusion of artifacts. The ROIs were selected to resemble the same areas selected using ChatGPT-4. Following that, texture features describing the first-order and second-order histograms were extracted from ROIs. The same feature extraction and logistic regression methodology described above was applied, allowing for a direct comparison of the 2 approaches.

#### Evaluating ChatGPT-4 for Imaging Findings–Based Diagnosis

**Figure 2:**
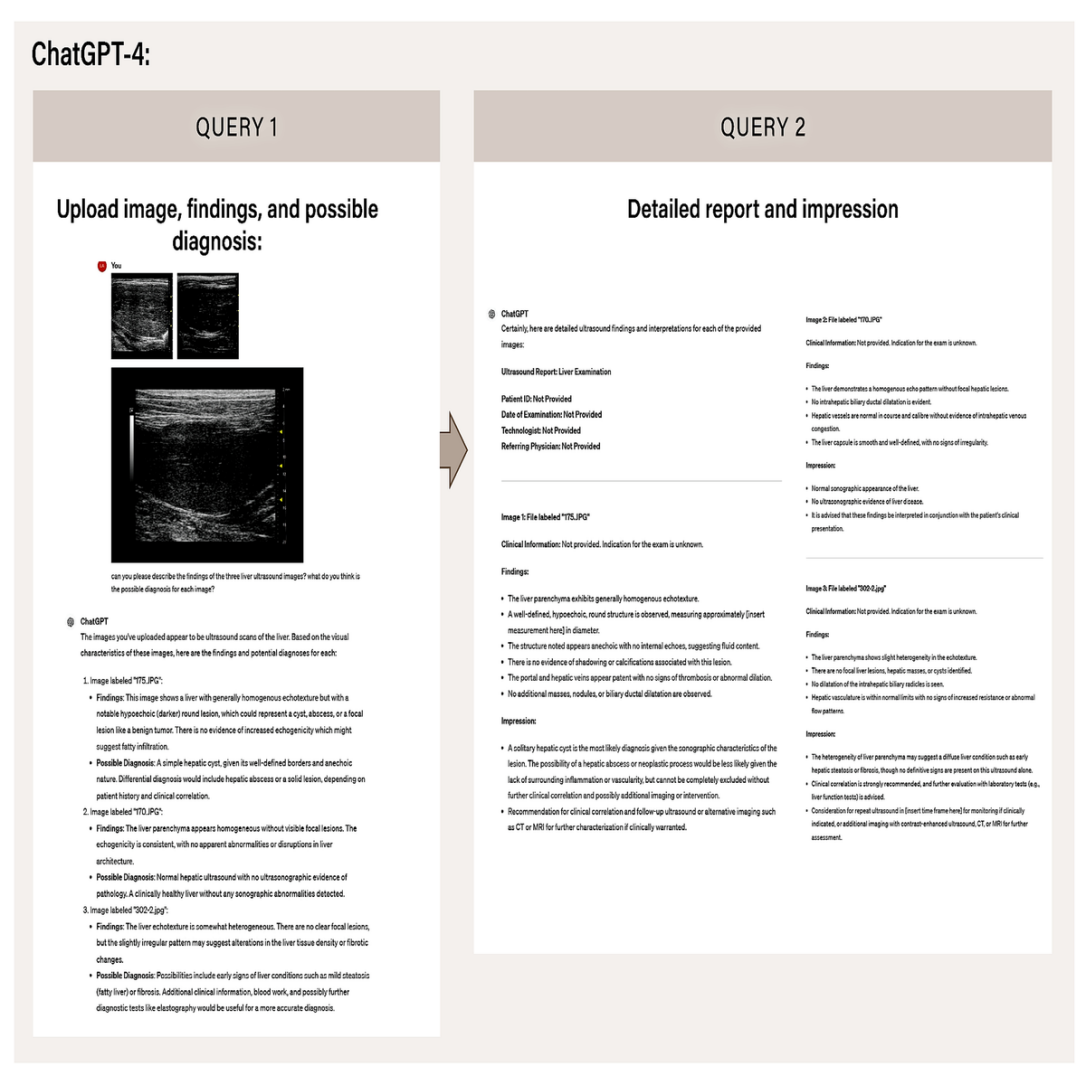
ChatGPT-4–assisted liver ultrasound image diagnosis and report generation workflow. The figure depicts the workflow of using ChatGPT-4 for generating liver ultrasound image findings, possible diagnoses, and detailed reports. In query 1, images are uploaded for analysis, and ChatGPT-4 provides initial findings and potential diagnoses based on visual characteristics, such as liver parenchyma echotexture and the presence of lesions. In query 2, ChatGPT-4 generates a detailed report and impression, summarizing the clinical interpretation of the ultrasound images. Each image is examined for hepatic abnormalities, including potential cysts, signs of fibrosis, or normal liver architecture, with impressions supporting clinical correlation or further diagnostic imaging recommendations. This stepwise approach demonstrates ChatGPT-4’s ability to assist in diagnostic interpretations and report generation for liver ultrasound studies, streamlining clinical workflows and enhancing diagnostic accuracy.

To explore the potential of ChatGPT-4 as a tool for distinguishing normal from abnormal liver cases, we conducted an experiment involving liver ultrasound images representing various conditions. We uploaded these images to ChatGPT-4 and tasked it with providing detailed descriptions of the findings and possible diagnoses for each image (Figure 2). ChatGPT-4’s output included comprehensive imaging findings that described the characteristics of the liver tissue and suggested potential diagnoses based on these observations.

Following this, we compared the diagnoses provided by ChatGPT-4 with the actual diagnoses to assess its diagnostic performance in identifying liver pathology. This involved calculating metrics such as sensitivity, specificity, and overall accuracy to determine how well ChatGPT-4 could identify normal and abnormal cases.

### Statistical Analysis

To interpret the differences in ultrasound texture features among the 3 liver health categories, we calculated the mean values and SEs. A 1-way ANOVA was conducted to identify any statistical differences across the study groups.

When comparing 2 groups, the Shapiro-Wilk test was used to assess normality. If the data did not meet the normality assumption, the Mann-Whitney test was applied to determine significance, otherwise, statistical significance was evaluated using 2-tailed paired Student *t* tests, with a threshold of *P*<.05.

Diagnostic performance of individual features, and combined, including sensitivity, specificity, accuracy, and *F*_1_-score were calculated. To support the visualization of multiclass separability, an additional exploratory analysis was performed using a decision tree classifier. A one-vs-rest classification scheme was used to generate ROC curves and compute AUC values for each class: fibrosis, normal, and steatosis. In addition, the intraclass correlation coefficient (ICC) analysis was performed between 2 observers to assess the reproducibility of ChatGPT-derived features. All analyses were performed using MedCalc software (version 19.0.5; MedCalc Software Ltd).

## Results

### Multiclass Liver Disease Classification by ChatGPT-4–Based Ultrasound Radiomics

#### Identification of Key Features

**Figure 3:**
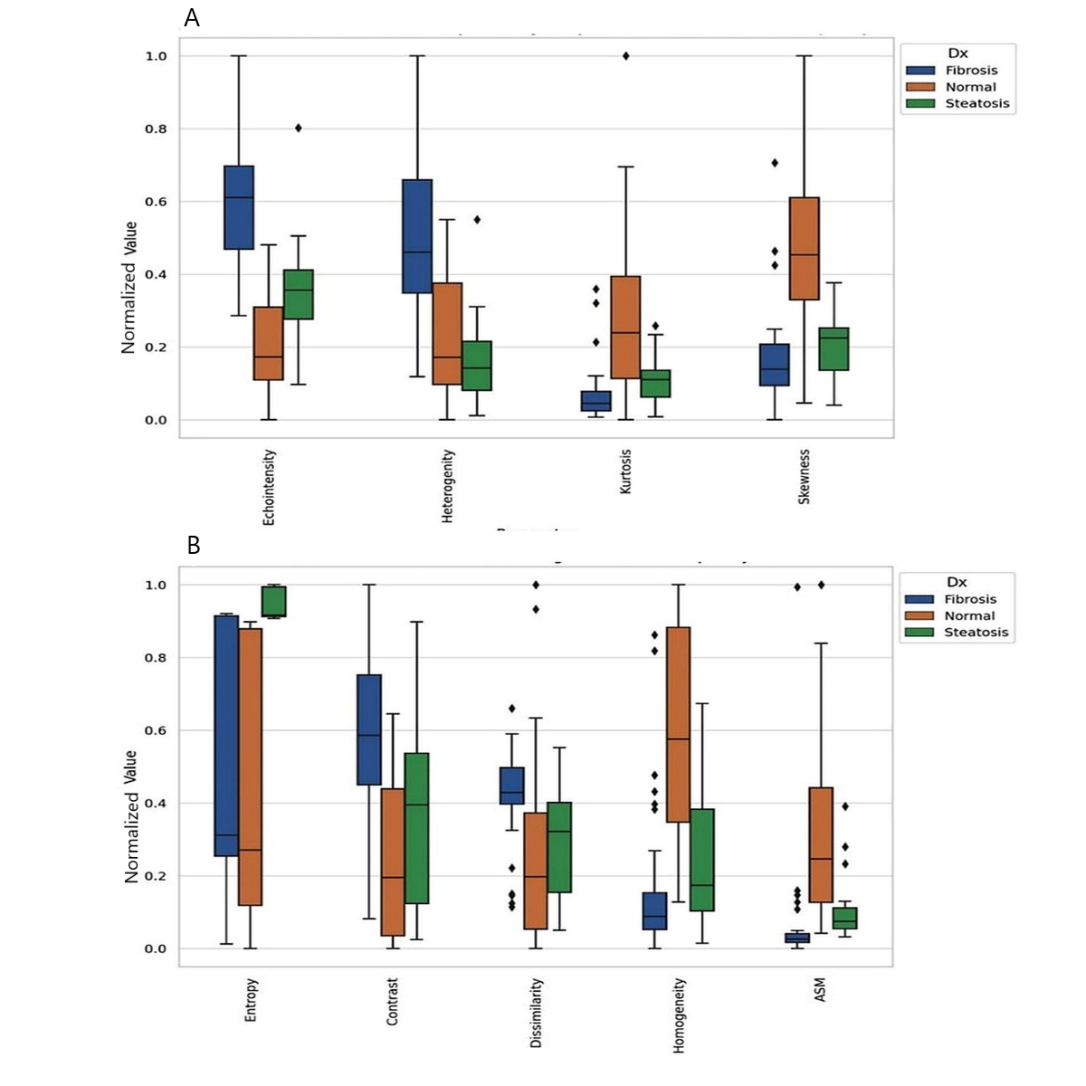
This figure presents the distribution of normalized texture features extracted from liver ultrasound images using ChatGPT-4, comparing 3 diagnostic groups: fibrosis, normal liver, and steatosis. (A) Plot 1 displays first-order histogram features, including echointensity, heterogeneity, kurtosis, and skewness. Fibrotic livers exhibit the highest echogenicity, followed by steatotic livers, both exceeding normal liver levels. Additionally, fibrosis is characterized by increased heterogeneity, whereas steatosis appears more homogeneous. (B) Plot 2 illustrates higher-order texture features, including entropy, contrast, dissimilarity, homogeneity, and ASM. Fibrosis is associated with greater contrast and dissimilarity, alongside reduced ASM, reflecting increased microstructural irregularity. Conversely, normal liver tissue demonstrates higher ASM and homogeneity, indicating a more uniform texture. ASM: angular second moment.

The ultrasound radiomics data processed by ChatGPT-4 has provided significant insights into the textural characteristics associated with various liver diseases (Figure 3). An ANOVA analysis identified 9 key textural features (from 10 features studied)—echo intensity, heterogeneity, skewness, kurtosis, contrast, homogeneity, dissimilarity, ASM, and entropy—as significantly varying among different liver conditions.

#### Predictive Performance of Individual Features

Further analysis revealed varying degrees of accuracy, sensitivity, and specificity for the identified imaging features across different metrics (Table 1). The accuracy of these features ranged from 0.48 to 0.62, with echointensity and entropy exhibiting the highest accuracy at 0.62. Specificity and sensitivity also varied, with echointensity showing a high specificity of 0.62 and entropy demonstrating a lower specificity at 0.42. Heterogeneity and skewness presented moderate accuracy levels at 0.57, with heterogeneity having slightly higher sensitivity. Energy stood out for its specificity at 0.66, while ASM, despite having the lowest sensitivity at 0.33, exhibited the highest specificity at 0.67. When these features were combined, the overall accuracy improved to 0.76, with a sensitivity of 0.83. An analysis of feature-wise *F*_1_-scores revealed also variability in their predictive contributions (Table 1). Echo intensity also exhibited the strongest performance (*F*_1_-score=0.85), while heterogeneity followed with *F*_1_-score of 0.67. Notably, the combined feature approach achieved *F*_1_-score (0.77), emphasizing the advantage of integrating multiple features, particularly weak ones.

**Table 1:** Performance metrics for key radiomic features in multiclass liver disease classification. Results are derived from logistic regression models configured as described above.

Feature	Accuracy	Sensitivity	Specificity	*F*_1_-score
Echo-intensity	0.62	0.56	0.62	0.85
Heterogeneity	0.57	0.50	0.55	0.67
Skewness	0.57	0.47	0.62	0.63
Kurtosis	0.48	0.36	0.64	0.63
ASM	0.48	0.33	0.67	0.42
Energy	0.57	0.47	0.66	0.42
Contrast	0.52	0.41	0.55	0.58
Dissimilarity	0.52	0.41	0.55	0.58
Entropy	0.62	0.60	0.42	0.56
Homogeneity	0.52	0.41	0.60	0.54

**Figure 4:**
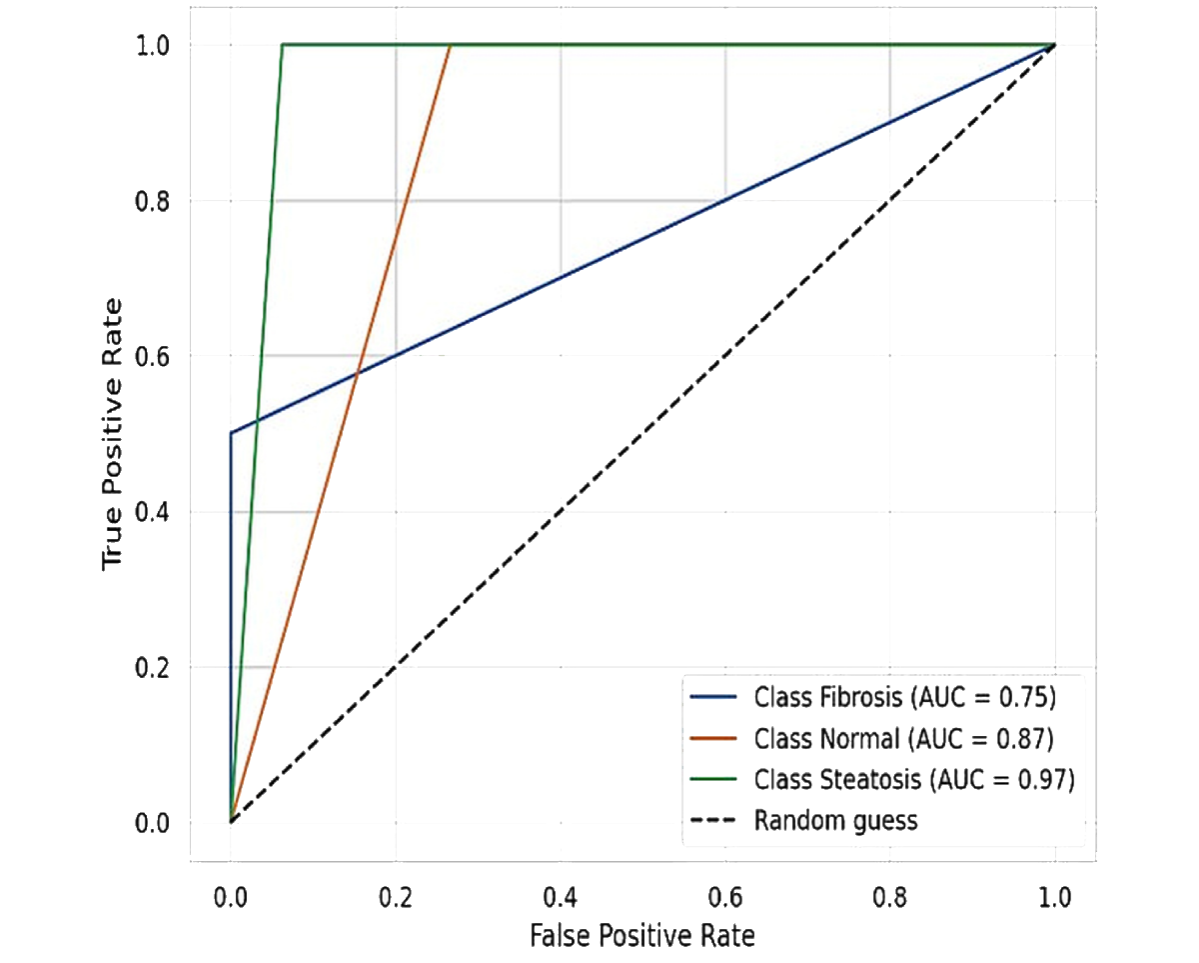
This ROC curve illustrates the diagnostic performance of ChatGPT-4 in classifying liver conditions using a decision tree model based on combined features. The model’s performance is evaluated across 3 classes: Fibrosis (the ROC curve for fibrosis shows an AUC [area under the ROC curve] of 0.75, indicating moderate diagnostic accuracy), Normal (the ROC curve for the normal class shows an AUC of 0.87, suggesting high diagnostic accuracy), and Steatosis (the ROC curve for steatosis shows an AUC of 0.97, indicating excellent diagnostic accuracy). The black dashed line represents a random guess with an AUC of 0.50. This figure demonstrates the capability of ChatGPT-4 to distinguish between different liver conditions with varying degrees of accuracy. ROC: receiver operating characteristic.

ROC curve analysis for features combined using a decision tree classifier showed the following AUC values: 0.75 for fibrosis, 0.87 for normal, and 0.97 for steatosis (Figure 4). ROC comparison for individual features is shown in Table 2 (Figure S1 in Multimedia Appendix 1). The comparison showed that echo intensity and heterogeneity were highest in fibrosis (0.91 and 0.86, respectively), suggesting increased structural disruption compared with steatosis and normal liver. ASM was highest in steatosis (0.88), reflecting greater textural uniformity, while fibrosis had the lowest value, indicative of higher heterogeneity. Contrast and dissimilarity, measures of local intensity variation, were most pronounced in fibrosis (0.79 and 0.73, respectively) and lowest in normal liver, reinforcing fibrosis’s greater textural complexity. Homogeneity and energy, which indicate texture smoothness and uniformity, were highest in normal liver (0.87 and 0.58, respectively), reflecting well-organized tissue architecture, and lowest in fibrosis, further supporting its structural disorganization.

**Table 2:** This table presents the area under the receiver operating characteristic (ROC) curve (AUC) values for radiomic features extracted from liver ultrasound images using ChatGPT-4, assessing their ability to differentiate fibrosis, steatosis, and normal liver tissue. These findings demonstrate the feasibility of ChatGPT-4–assisted ultrasound radiomics for noninvasive liver disease characterization.

	Fibrosis	Steatosis	Normal
Echo intensity	0.91	0.39	0.12
Heterogeneity	0.86	0.33	0.23
Kurtosis	0.22	0.51	0.82
Skewness	0.22	0.45	0.87
Angular second momentum	0.11	0.88	0.59
Correlation	0.53	0.33	0.61
Dissimilarity	0.73	0.39	0.33
Contrast	0.79	0.42	0.22
Entropy	0.44	0.90	0.21
Energy	0.49	0.43	0.58
Homogeneity	0.20	0.49	0.87

#### Reproducibility and Reliability

To assess the reproducibility of ChatGPT-4 outputs across users, 2 independent observers used the same ChatGPT-4–assisted workflow to select ROIs and extract radiomic features from the same ultrasound images. Both were trained physicians with clinical and research expertise in liver ultrasound. The ICC was calculated across the extracted radiomic features to quantify consistency. The results demonstrated high reproducibility for most features exceeding ICC of 0.8, with energy (ICC=0.96), correlation (ICC=0.92), and echo intensity (ICC=0.88) showing excellent agreement between observers (Table 3). Entropy (ICC=0.81) and homogeneity (ICC=0.81) also indicated strong reliability, suggesting consistent feature extraction across different evaluators. Skewness (ICC=0.6) exhibited moderate agreement, while ASM showed the lowest ICC (ICC=0.25), indicating poor reproducibility for this metric.

**Table 3:** This table presents the intraclass correlation coefficients (ICC) assessing interobserver agreement for key radiomic features extracted from liver ultrasound images using ChatGPT-4. These results indicate strong to excellent reliability for most features, supporting the robustness of ChatGPT-4–assisted radiomic analysis in liver ultrasound imaging.

Feature	ICC
Echo-intensity	0.88
Heterogeneity	0.90
Kurtosis	0.39
Skewness	0.60
Angular second momentum	0.25
Correlation	0.92
Dissimilarity	0.78
Contrast	0.89
Entropy	0.81
Energy	0.96
Homogeneity	0.81

#### Binary Classification of Healthy Liver Versus Steatosis and Fibrosis Using ChatGPT-4 Ultrasound Radiomics

Significant distinctions were observed between normal liver and diseased conditions, particularly in 8 out of 10 analyzed features. For the binary comparison between normal and liver diseases (steatosis and fibrosis), 8 features showed significant differences (<0.05): echo-intensity (27.47 vs 50.47), heterogeneity (423.96 vs 687.17), skewness (0.95 vs 1.86), kurtosis (1.34 vs 5.24), energy (0.18 vs 0.22), contrast (30.65 vs 57.37), ASM (0.003 vs 0.001), and homogeneity (0.20 vs 0.38), for normal versus liver disease, respectively.

Comparing normal to fibrosis revealed significant differences in 8 features (<0.05): echo-intensity, heterogeneity, skewness, kurtosis, entropy, contrast, homogeneity, and correlation. For normal versus steatosis, 6 features showed significant differences: echo-intensity, entropy, skewness, kurtosis, Homogeneity, ASM, and energy. These mean values for the features are summarized in Table 4.

**Table 4:** This table illustrates the differences between liver disease groups (normal, steatosis, and fibrosis) by showing the mean values of features extracted through ChatGPT-4–based radiomics analysis. The features include echo intensity, heterogeneity, skewness, kurtosis, contrast, homogeneity, dissimilarity, angular second momentum (ASM), and entropy. The mean values for these features provide insights into the distinct textural characteristics associated with each liver disease group.

	Echo-intensity, mean (SD)	Heterogeneity, mean (SD)	Entropy, mean (SD)	Skewness, mean (SD)	Kurtosis, mean (SD)	Energy, mean (SD)	Contrast, mean (SD)	Dissimilarity, mean (SD)	Homogeneity, mean (SD)	ASM, mean (SD)
Liver disease (fibrosis and steatosis)	50.47 (2.34)	687.17 (57.19)	8.91 (0.28)	0.95 (0.05)	1.34 0.20	0.18 (0.03)	57.37 (4.31)	5.73 (0.26)	0.20 (0.01)	0.001 (0.00)
Liver fibrosis	56.67 (2.56)	857.07 (65.62)	7.99 (0.34)	0.93 (0.07)	1.18 (0.27)	0.18 (0.03)	64.65 (5.04)	6.21 (0.29)	0.19 (0.02)	0.001 (0.00)
Liver steatosis	39.10 (2.88)	366.24 (46.85)	10.66 (0.06)	0.97 (0.06)	1.64 (0.29)	0.19 (0.05)	43.62 (6.71)	4.81 (0.43)	0.22 (0.02)	0.001 (0.00)
Normal	27.44 (2.32)	423.96 (57.93)	8.15 (0.46)	1.86 (0.15)	5.24 (0.97)	0.22 (0.03)	30.65 (5.22)	4.88 (0.77)	0.38 (0.03)	0.003 (0.00)

#### Distinguishing Liver Disease by ChatGPT-4–Based Ultrasound Image Findings

The classification tool for liver ultrasound images exhibited strong diagnostic performance across 3 categories: normal liver, fibrosis, and steatosis. Achieving an overall accuracy of 77%, the tool demonstrated its potential in aiding radiological assessments. For normal liver conditions, the tool achieved a precision, recall, and *F*_1_-score of 0.75, indicating reliable detection accuracy. In the case of fibrosis, the tool excelled with a perfect recall of 1.00, meaning it successfully identified all fibrosis cases, and an *F*_1_-score of 0.86, with a precision of 0.75. This highlights its robustness in diagnosing fibrotic conditions without missing any positive cases. However, for steatosis, while the tool showed a high precision of 0.80, the recall was slightly lower at 0.67, leading to an *F*_1_-score of 0.73. This indicates a strong ability to correctly identify steatosis when predicted, though there is room for improvement in sensitivity.

The macroaveraged metrics (precision=0.77, recall=0.81, and *F*_1_-score=0.78) and weighted averages (precision=0.77, recall=0.77, and *F*_1_-score=0.76) further underscore the tool’s balanced performance across different liver conditions. These results suggest that while the tool is already valuable for distinguishing normal and abnormal liver conditions, further refinements could enhance its sensitivity, particularly for steatosis.

### Evaluation of IDL-Based Ultrasound Radiomics for Liver Disease Classification

#### Identification of Key Textural Features

**Figure 5:**
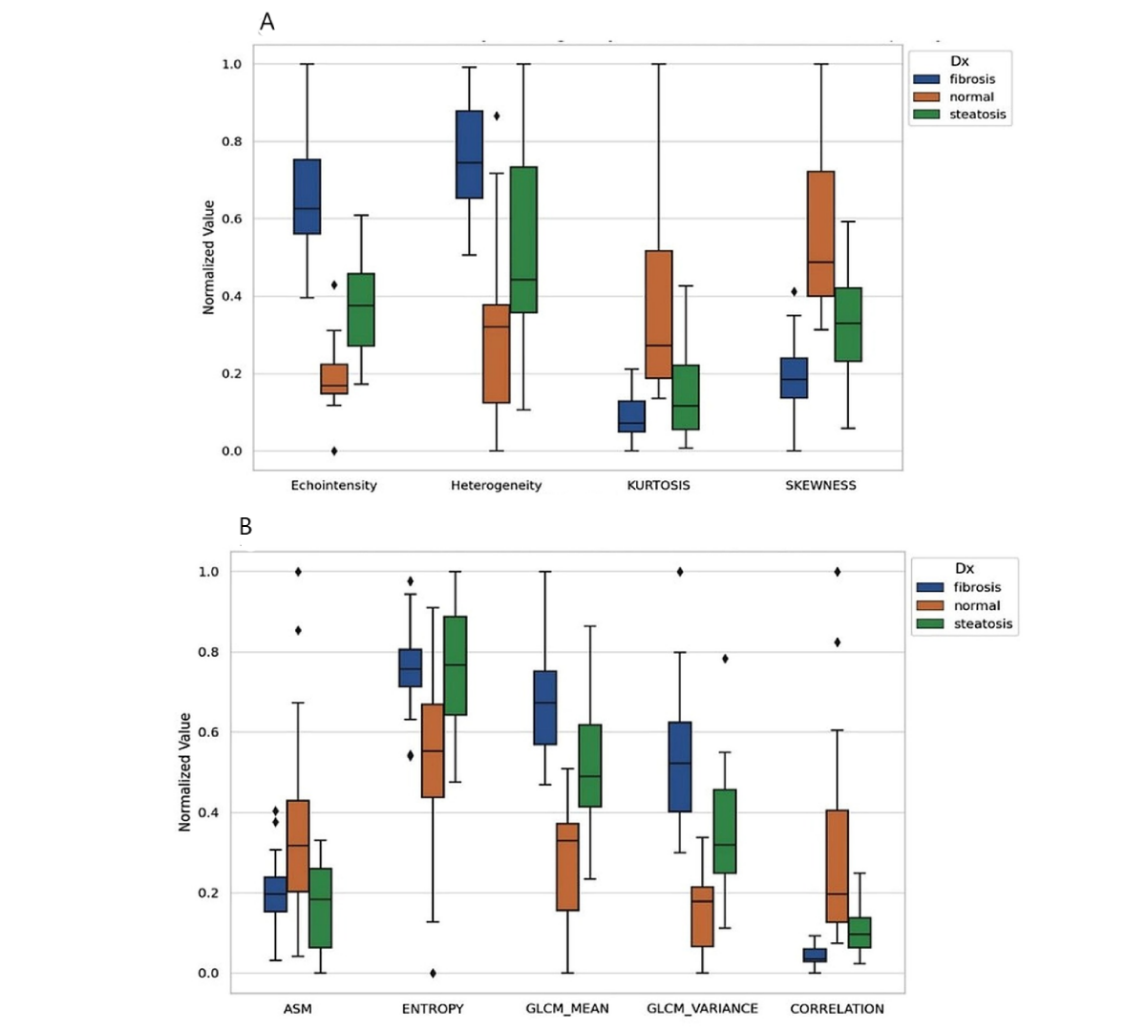
Interactive data language (IDL)–based radiomics analysis in liver ultrasound images: This figure presents the distribution of texture parameters extracted using IDL from liver ultrasound images, comparing 3 diagnostic groups: fibrosis (blue), normal liver (orange), and steatosis (green). (A) Plot displays first-order texture features, including echointensity, heterogeneity, kurtosis, and skewness. Fibrotic livers exhibit increased echogenicity and heterogeneity compared with both normal and steatotic livers, reflecting structural alterations associated with fibrosis. (B) Plot illustrates higher-order texture features, including ASM, entropy, GLCM mean, GLCM variance, and correlation. Fibrotic livers demonstrate higher GLCM mean and variance, indicating greater textural complexity, whereas normal liver tissue exhibits lower values for these parameters but higher ASM and correlation, suggesting a more homogeneous texture. These findings highlight the capability of IDL-based radiomics in quantifying microstructural liver alterations across different pathological states, reinforcing its potential as an advanced imaging biomarker for disease characterization. ASM: angular second moment; GLCM: gray-level co-occurrence matrix.

The radiomics analysis of liver ultrasound images conducted using IDL has provided significant insights into the textural characteristics associated with various liver diseases. Through ANOVA analysis, 9 textural features were identified as significantly varying among groups with different liver conditions (Figure 5).

#### Predictive Performance of Features

The predictive performance of features of these ultrasound imaging features varied, with accuracy ranging from 0.47 to 0.76 sensitivity, from 0.33 to 0.73, and specificity from 0.43 to 0.70 (Table 5). The feature “echo-intensity” demonstrated the highest performance with an accuracy of 0.76, sensitivity of 0.73, and specificity of 0.53, indicating balanced performance. Similarly, “Heterogeneity” also showed an accuracy of 0.76, with a sensitivity of 0.68 and a specificity of 0.51. On the other hand, “Kurtosis” had lower accuracy at 0.48 and sensitivity at 0.38, but a higher specificity of 0.64, highlighting its strength in correctly identifying true negative cases. Integrating multiple textural features enhances diagnostic performance. By combining the features, the overall accuracy improved to 0.77, with a notable accuracy of 0.89. Similarly, *F*_1_-score performance varied across features, with echo intensity achieving the highest *F*_1_-score (0.84), indicating its superior predictive power. Heterogeneity also performed well, with an *F*_1_-score of 0.56. In contrast, kurtosis, ASM, entropy, and correlation had the lowest *F*_1_-scores (ranging from 0.26), reflecting weaker predictive contributions. Notably, the combined feature approach achieved the highest *F*_1_-score (0.81), emphasizing the advantage of integrating multiple features to enhance predictive accuracy.

**Table 5:** Diagnostic accuracy and performance of radiomic features extracted using interactive data language software. This table displays the diagnostic accuracy and performance metrics for various textural features extracted from the liver ultrasound images using interactive data language software as part of liver texture analysis. These metrics provide insights into the effectiveness of each feature in distinguishing between different liver conditions, contributing to the overall assessment of liver disease.

Feature	Accuracy	Sensitivity	Specificity	*F*_1_-score
Echo-intensity	0.76	0.73	0.53	0.84
Heterogeneity	0.76	0.68	0.51	0.56
Kurtosis	0.47	0. 38	0.64	0.38
Skewness	0.67	0.58	0.55	0.48
ASM^a^	0.48	0.33	0.56	0.26
Entropy	0.48	0.33	0.43	0.26
GLCM^b^_mean	0.67	0.56	0.51	0.48
GLCM_variance	0.62	0.59	0.53	0.57
Correlation	0.48	0.33	0.7	0.26

^a^ASM: angular second momentum.

^b^GLCM: gray-level co-occurrence matrix.

### Comparison Between ChatGPT and IDL Features

#### Correlation and Agreement Analysis Between Feature Sets

**Figure 6:**
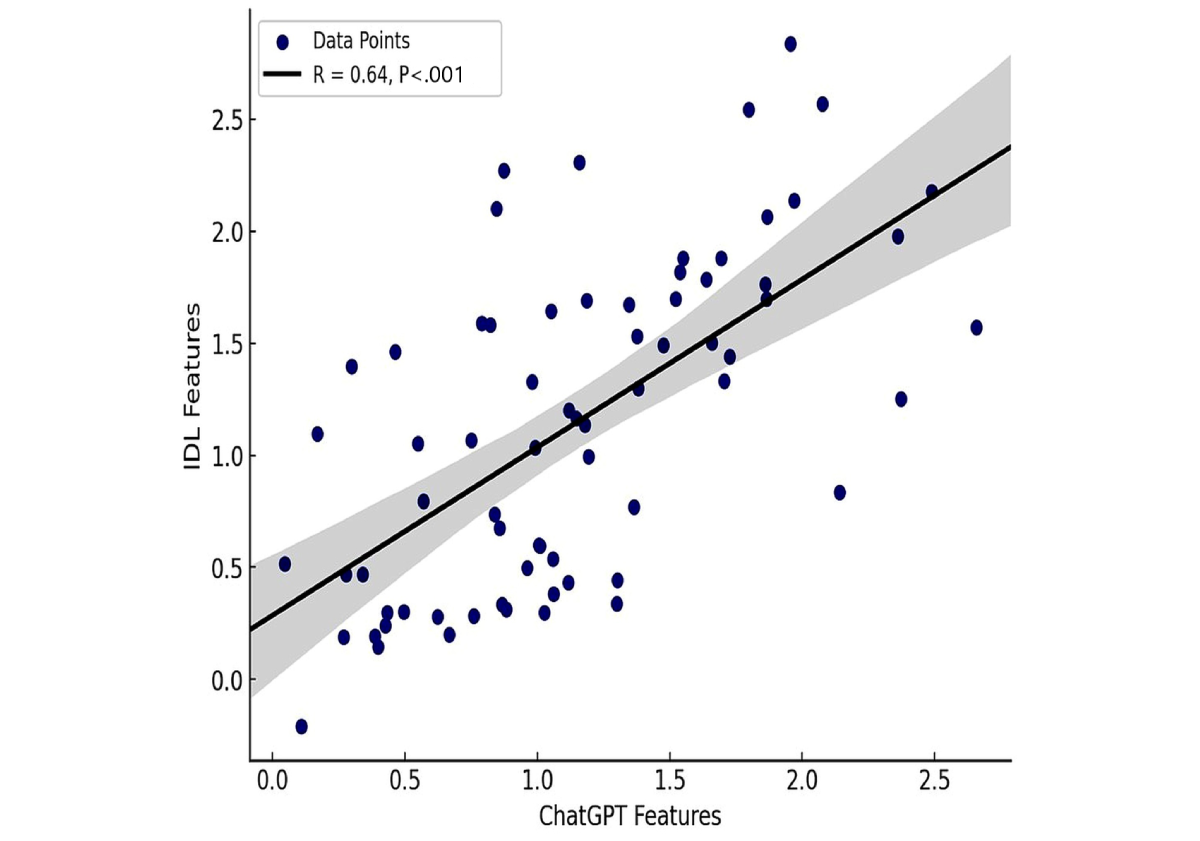
Correlation between ChatGPT Features and IDL Features. The scatter plot illustrates the relationship between ChatGPT features and IDL features, with a Pearson correlation coefficient (r) of 0.64 and a significant *P* value (*P*<.001). Each blue circle represents an individual data point, while the solid black line shows the fitted linear regression model. The shaded region surrounding the regression line represents the 95% CI. The moderate positive correlation suggests that as ChatGPT features increase, IDL features tend to increase as well, indicating a consistent, albeit not perfect, relationship between the 2 feature sets. IDL: interactive data language.

To assess the relationship between ChatGPT-4-derived features and IDL-based features, we performed correlation and agreement analyses. Each feature set was consolidated into a single value using multiple regression, allowing for a direct, one-to-one comparison between the 2 methods. The multiple linear regression model using ordinary least squares was applied to combine all extracted radiomic features into a single predicted value per image, with the liver disease category as the dependent variable. This was done separately for ChatGPT-4 and IDL outputs to generate comparable summary values. The results showed a moderate positive correlation (*r*=0.64) across all extracted features, which was statistically significant (*P*<.001; Figure 6). In other words, increases in ChatGPT-4 feature values tended to coincide with increases in IDL feature values, albeit with some variability. While this correlation is not perfect, it demonstrates that ChatGPT-4–derived features are reasonably well aligned with IDL features, supporting the feasibility of ChatGPT-4 for ultrasound radiomics analysis.

**Figure 7:**
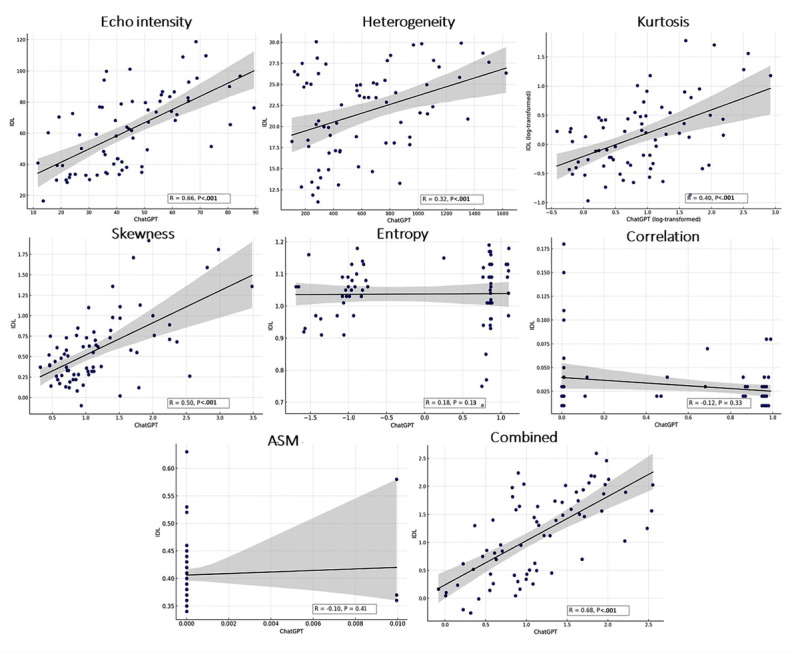
This figure presents scatter plots illustrating the correlation between radiomic features extracted using ChatGPT-4 and the corresponding common features derived from the reference software, IDL. Each subplot represents a specific feature, with ChatGPT-4 values on the x-axis and IDL values on the y-axis. Linear regression lines with shaded 95% CIs are shown to illustrate the strength and direction of the associations. Pearson correlation coefficients (r) and *P* values (P) are reported for each feature. Strong correlations were observed for echo intensity (r=0.66, *P*<.001) and skewness (r=0.50, *P*<.001), while entropy (r=0.18, *P*=.13), correlation (r=–0.12, *P*=.33), and ASM (r=–0.10, *P*=.41) showed weaker or nonsignificant associations. The final plot displays a combined score derived from the 7 shared features, generated using multiple regression. This aggregated output demonstrated a moderate correlation (r=0.68) between ChatGPT-4 and IDL, supporting overall agreement across platforms. These findings highlight both the variability in feature-level agreement and the potential value of composite feature models in radiomics analysis. ASM: angular second momentum; IDL: interactive data language.

We further examined the correlation between the 2 software packages by focusing on 7 common features extracted by ChatGPT-4 and IDL showing a correlation (*r*) of 0.68 (Figure 7). The degree of correlation varied among the individual features, with the strongest correlation observed for combined features (*r*=0.68, *P*<.001). Notably, first-order histogram measures such as echo-intensity (*r*=0.60) and kurtosis (*r*=0.52) showed stronger correlations, whereas GLCM-based features exhibited weaker alignment. In particular, measures like correlation and kurtosis derived from GLCM demonstrated lower correlations.

To further assess agreement between features extracted by the 2 software, a Bland-Altman analysis was performed (Figure S2 in Multimedia Appendix 1). The results demonstrated that combined features exhibited the best agreement, with narrow agreement limits and minimal bias, reinforcing their robustness. Skewness showed particularly strong agreement, indicating interchangeability between ChatGPT-4 and IDL for this feature. Minimal proportional bias was observed for well-correlated features, supporting the feasibility of using ChatGPT-4 for radiomics analysis in this context. Based on these results, the agreement between ChatGPT-4 and IDL-derived features can be categorized into three levels: (1) strong agreement (reliable and interchangeable): skewness and correlation; (2) moderate agreement (requires minor adjustments): ASM, entropy, and echo-intensity; and (3) weak agreement (fundamental differences requiring major corrections): kurtosis and heterogeneity.

#### Processing Time Comparison

In addition to feature correlation, we also compared batch processing efficiency between ChatGPT-4 and IDL-based tools. The results demonstrated that ChatGPT-4 outperformed IDL in processing speed. ChatGPT-4 completed the entire analysis process—including ROI selection, refinement, and texture analysis—in 4 minutes and 12 seconds for a batch of 10 images (the maximum batch size). In contrast, IDL required approximately 50 seconds per case, totaling over 8 minutes for the same batch. ChatGPT, therefore, showed more than a 40% reduction in processing time, highlighting ChatGPT-4’s efficiency in automated batch processing. These findings suggest that ChatGPT-4 provides a viable alternative for high-throughput ultrasound radiomics analysis, offering both speed and reasonable alignment with IDL-based feature extraction.

## Discussion

### Expanding ChatGPT-4’s Role in Radiology

AI and natural language processing tools, such as ChatGPT, have been increasingly explored for their role in enhancing radiology workflows [39]. Recent studies demonstrated how ChatGPT can be integrated into radiology workflows to improve efficiency in patient registration, scheduling, image acquisition, interpretation, and reporting [40,41]. The findings of these studies highlight ChatGPT’s potential to streamline repetitive tasks, reduce radiologist workload, and enhance communication in diagnostic imaging. Our study builds upon this foundation by extending the role of ChatGPT-4 beyond workflow optimization into advanced radiomics analysis. Specifically, we evaluate ChatGPT-4’s ability to extract quantitative ultrasound texture features, distinguish between different liver disease states, and compare its performance against conventional radiomics software. By bridging workflow optimization with diagnostic analysis, our findings contribute to the ongoing evolution of AI-assisted radiology, reinforcing ChatGPT’s potential as a tool for both administrative and analytical applications in medical imaging.

### ChatGPT-4’s Diagnostic Performance and Reproducibility

Our study results show that ChatGPT-4’s radiomic analysis exhibited robust performance in distinguishing among the 3 liver pathology groups, achieving a sensitivity of 0.83 and AUC exceeding 0.75, when all radiomic features were combined. While the diagnostic utility of individual features varied, the aggregated analysis compensated for weaker predictors, thereby enhancing overall classification accuracy. Moreover, the high ICC values observed between independent observers (reaching 0.92) suggest excellent reproducibility, reinforcing the robustness of ChatGPT-4–derived texture parameters in ultrasound imaging. However, not all features demonstrated high reliability; for instance, ASM yielded an ICC of 0.25, indicating poor reproducibility. Such discrepancies in intraclass agreement for extracted ultrasound features can arise from small differences in ROI placement, even if they are close. Ultrasound images are highly sensitive to pixel-level changes, which can affect texture-based features. Additionally, interpolation effects, quantization errors, and software implementation variability can contribute to differences. These findings underscore the need for further refinement in feature extraction methodologies, particularly for features with lower reproducibility. Future research should prioritize the standardization of algorithms to enhance observer consistency, ensuring that AI-generated radiomic features are both reliable and clinically actionable.

### Interpretation of the Radiomic Biomarkers in Liver Disease

The radiomic biomarkers identified in this study align with established pathophysiological changes in liver disease. Increased heterogeneity and entropy, for instance, reflect greater structural disorder where excessive collagen deposition disrupts tissue uniformity, consistent with fibrosis [42,43]. GLCM-based texture features provide additional microstructural insights, for example, ASM (or energy) serving as an index of texture uniformity—higher values indicate preserved architecture, while lower values suggest structural disruption, such as that seen in fibrosis [44]. These features may serve as robust, noninvasive biomarkers for disease detection and monitoring. Our results showed that distinct textural patterns can be related to different liver conditions. Fibrosis presents with increased echogenicity, heterogeneity, and contrast, indicating architectural disruption. While steatosis also exhibits high echogenicity, it is associated with a smoother, more homogeneous texture, suggesting uniform yet structurally altered tissue. In contrast, normal liver maintains the most uniform texture, with high homogeneity and low contrast, reflecting preserved tissue organization.

### Comparison of ChatGPT-4 With Traditional Image Analysis Software

Direct comparison between features extracted by ChatGPT-4 and IDL revealed a moderate correlation (*r*=0.68), when features are combined with notable variations between specific features on an individual basis. First-order features, which primarily assess pixel intensity distributions, exhibited strong agreement between the 2 platforms, whereas GLCM-based features showed greater discrepancies. This discrepancy is likely attributable to differences in pixel adjacency definitions, quantization methods, and sampling protocols across the 2 analytical frameworks. These results highlight a persistent challenge in radiomics: reproducibility across different software implementations. Variability in image acquisition parameters, preprocessing steps, and computational feature extraction methodologies can significantly impact radiomic feature consistency. Prior studies have underscored the necessity of harmonized radiomic pipelines to enhance cross-platform reproducibility [45,46]. Establishing standardized radiomic workflows will be critical for ensuring the clinical applicability of AI-driven ultrasound analysis.

A key advantage of ChatGPT-4 in this study was its ability to process multiple images in parallel, demonstrating significant efficiency gains over conventional software. Notably, processing time was reduced by more than 40% compared with IDL, suggesting that AI-driven tools can significantly enhance radiological workflow efficiency. This capability is particularly valuable in research settings requiring high-throughput image analysis, as well as in clinical environments where real-time assessment is essential for guiding interventional procedures. Moreover, ChatGPT-4’s scalability supports its application in large-scale imaging studies, enabling rapid dataset processing while minimizing manual input. This efficiency could facilitate applications in population-based screening programs, multicenter trials, and AI-assisted educational platforms. While compute capacity was controlled for in this study, we acknowledge that hardware variability can influence software performance. Future work should evaluate AI efficiency across diverse computing environments to better account for system-dependent constraints. Despite its slightly lower diagnostic performance compared with IDL, the results are encouraging given that ChatGPT-4 was not originally designed for medical image analysis. With additional domain-specific training and fine-tuning using large-scale ultrasound datasets, its performance is expected to improve. Future research should explore ChatGPT-4’s integration into routine radiology workflows, particularly in triage settings, where automated interpretation of liver ultrasound images could expedite clinical decision-making and optimize resource allocation.

### Limitations and Challenges

#### Session Variability and Model Robustness

Despite its promising performance, ChatGPT-4 exhibited session-dependent variability in feature extraction. This phenomenon, which possibly arises from differences in how the model processes context and maintains internal states across separate analyses, introduces potential inconsistencies in feature reproducibility. While batch analyses remained stable, independent session resets occasionally yielded variations in extracted parameters. Session-dependent variability is a recognized limitation of large language models [47-49] and warrants further investigation in the context of medical imaging. To mitigate this challenge, we refined our prompting strategies, ensuring that feature extraction parameters were explicitly aligned across sessions. While steps were taken to standardize ChatGPT-4 prompts and maintain session continuity, variability in output due to the model’s inherent stochastic nature remains a limitation. Although incomplete feature sets were addressed through repeated prompting and prompt refinement, future studies may also benefit from averaging outputs across multiple runs or sessions to account for variability and enhance consistency. Additionally, future research should prioritize the development of standardized initialization protocols and structured prompt engineering strategies to improve the reproducibility of AI-driven radiomic analyses.

#### Automated ROI Selection

A key limitation of ChatGPT-4 is its fully automated ROI selection, which lacks the flexibility and precision needed for clinical applications. This may affect diagnostic accuracy, especially when critical pathological features fall outside the AI-defined ROI. While ChatGPT-4 does not allow direct manual ROI adjustments, we used a hybrid approach [50], iteratively refining prompts to guide the model until the desired ROI was accurately identified. This method combined AI-driven automation with user oversight, improving ROI placement and reducing errors. Future iterations of ChatGPT-4 could enhance clinical applicability by incorporating interactive manual ROI modifications [51]. Additionally, integrating advanced machine learning algorithms could refine automated ROI selection, allowing AI to prioritize clinically relevant areas [52]. A promising direction is the development of hybrid models that preselect an ROI while allowing clinician refinement, balancing automation with expert oversight [53].

#### Preclinical Model and Clinical Translatability

Our preclinical liver disease model closely mirrors human pathology, with histological findings aligning well with clinical presentations of fibrosis and steatosis. This translatability strengthens the relevance of our results; the model has undergone extensive validation to ensure robustness and suitability for studying liver disease [30-32]. Nonetheless, this study serves as an initial assessment of ChatGPT-4 in a preclinical setting. Future work will extend to human liver ultrasound datasets, potentially involving diverse populations and multiple medical centers to enhance generalizability. Importantly, moving to clinical datasets raises privacy and ethical concerns, requiring strict compliance with HIPAA (Health Insurance Portability and Accountability Act), GDPR (General Data Protection Regulation), and other data security frameworks. Additionally, AI bias—stemming from skewed or nonrepresentative training data—remains a critical challenge, necessitating multicenter validation to ensure fairness and accuracy across varied clinical settings.

#### Uncertainty in AI-Generated Diagnoses

Reliable AI outputs are critical in medical imaging. Currently, ChatGPT-4 lacks inherent uncertainty quantification. Integrating probabilistic methods could improve reliability by assigning confidence levels based on prior data distributions, similar to deep learning–based radiomics [54]. Monte Carlo dropout modeling could provide uncertainty intervals, flagging cases needing further review [55]. Ensemble modeling could further enhance reliability through consensus-based confidence scores [56]. Explainability improvements, such as structured reasoning frameworks, would support informed decision-making [57]. Implementing these methods would ensure ChatGPT-4 functions as a decision-support tool rather than an autonomous diagnostic system.

### Clinical Applications and Future Directions

This study highlights ChatGPT-4’s potential in medical imaging, particularly in image interpretation. While CNNs have achieved over 90% accuracy in tasks like liver fibrosis staging [44,58], ChatGPT-4 offers distinct advantages by integrating image analysis with narrative generation and enabling interactive ROI refinement [19]. Unlike traditional deep learning models requiring extensive training, ChatGPT-4’s adaptability supports multimodal integration, making it a promising tool for clinical applications. Fine-tuning on specialized datasets could enhance its diagnostic accuracy, bridging the gap between specialized AI models and broader usability. Enhancing ChatGPT-4’s clinical utility involves several advancements. Transfer learning can improve domain-specific accuracy by incorporating structured radiology reports and labeled diagnostic cases [59]. Multimodal training could allow it to analyze medical images alongside textual and radiomic data, improving correlation with clinical insights [60]. Real-time clinical decision support through interactive learning could refine outputs, while integrating longitudinal patient data could enhance disease monitoring, particularly for chronic conditions [61]. Future research should compare ChatGPT-4 with leading deep learning models to evaluate its role in multimodal medical imaging.

Integrating ChatGPT-4 into clinical workflows has the potential to enhance diagnostic efficiency by streamlining triage, anomaly detection, and preliminary report generation. AI-driven tools have demonstrated the ability to expedite time-to-diagnosis by prioritizing critical imaging findings [1,19]. In liver ultrasound, ChatGPT-4 could assist by distinguishing normal scans or minor abnormalities, allowing radiologists to focus on complex cases. Its radiomic analysis capabilities may facilitate early disease detection, akin to AI models that have identified microvascular changes in brain imaging and tumor margins in mammography [5,7]. Automated report generation is another promising application, as AI-generated reports have been shown to match human interpretation in accuracy [20,21]. Additionally, real-time feedback during ultrasound-guided procedures and batch-processing for large-scale imaging analysis could support multicenter studies and population-based disease screening [11,26]. Despite its potential, key challenges include validation across diverse populations, regulatory approval, and clinician training. Prospective studies are needed to assess ChatGPT-4’s impact on diagnostic accuracy, workflow efficiency, and patient outcomes. Addressing these challenges could establish ChatGPT-4 as a transformative tool in radiology, optimizing early disease detection and clinical workflows.

### Conclusions

In conclusion, our study confirms the feasibility of using ChatGPT-4 for liver disease diagnosis through ultrasound image analysis, emphasizing its potential to assist radiologists in making more accurate diagnoses. Despite some limitations, ChatGPT-4's ability to efficiently handle large-scale image datasets and its robust feature extraction capabilities make it a valuable tool for enhancing diagnostic accuracy and supporting radiological decision-making. By integrating ChatGPT-4 into radiological workflows, radiologists can leverage its capabilities to improve the precision and efficiency of liver ultrasound image analysis. This tool's potential to manage vast amounts of data with high efficiency is particularly appealing in modern medical research and clinical practice.

## Appendix

Multimedia Appendix 1 Additional figures and tables.

## Abbreviations

AI: artificial intelligence
ASM: angular second momentum
AUC: area under the receiver operating characteristic curve
GDPR: General Data Protection Regulation
GLCM: gray-level co-occurrence matrix
HIPAA: Health Insurance Portability and Accountability Act
ICC: intraclass correlation coefficient
IDL: interactive data language
ROC: receiver operating characteristic
ROI: region of interest
