# Supplementary material for: ChatGPT-4–Driven Liver Ultrasound Radiomics Analysis: Diagnostic Value and Drawbacks in a Comparative Study
Source: JMIR AI. 2025 Jun 30;4:e68144. doi: 10.2196/68144 (PMC12260471; doi:10.2196/68144)
Supplement: Multimedia Appendix 1 [file ai_v4i1e68144_app1.docx]

**Table S1.** This table provides representative examples of the standardized prompts used to guide ChatGPT-4 during each step of the liver ultrasound radiomics workflow. Prompts were carefully phrased to ensure consistent ROI selection and feature extraction across all images. These prompts were applied in each session to minimize variability and maintain reproducibility in the semi-automated analysis pipeline.

| Analysis Step | Prompt Example |
| --- | --- |
| Image Preparation | Please upload the liver ultrasound image(s) for analysis. |
| ROI Definition | Select a centrally located region of interest (ROI) within the liver parenchyma, avoiding major vessels and acoustic shadows. |
| ROI Confirmation | Display the selected ROI and confirm its location before proceeding with feature extraction. |
| ROI Adjustment | Please adjust the ROI to the correct position. Please move the ROI slightly to the right/left/up/down. |
| Feature Extraction | Extract texture features from the ROI, including echo intensity, heterogeneity, skewness, kurtosis, energy, entropy, contrast, homogeneity, dissimilarity, and angular second moment. |
| Batch Processing Setup | Apply the same ROI placement and feature extraction steps to all uploaded liver ultrasound images. |
| Output Display | Summarize and report the extracted texture features with clear labels for each image. |

**Table S2.** This table illustrates the stratified allocation of 70 cases into training (60%), testing (20%), and validation (20%) cohorts for the development and evaluation of a logistic regression-based diagnostic model utilizing radiomic features from liver ultrasound images. Stratified random sampling was employed to ensure balanced representation across liver disease categories. The training set facilitated model development, the testing set provided an initial performance assessment, and the validation set enabled independent evaluation of generalizability. This structured approach ensures methodological rigor and mitigates potential biases, reinforcing the model’s clinical applicability and translational relevance.


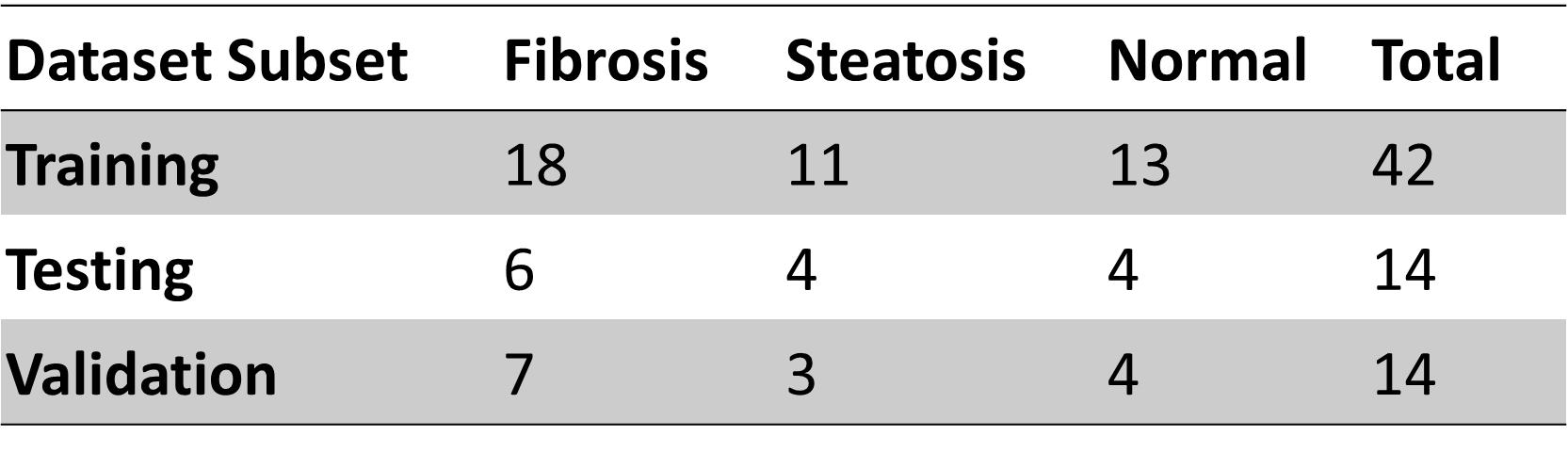


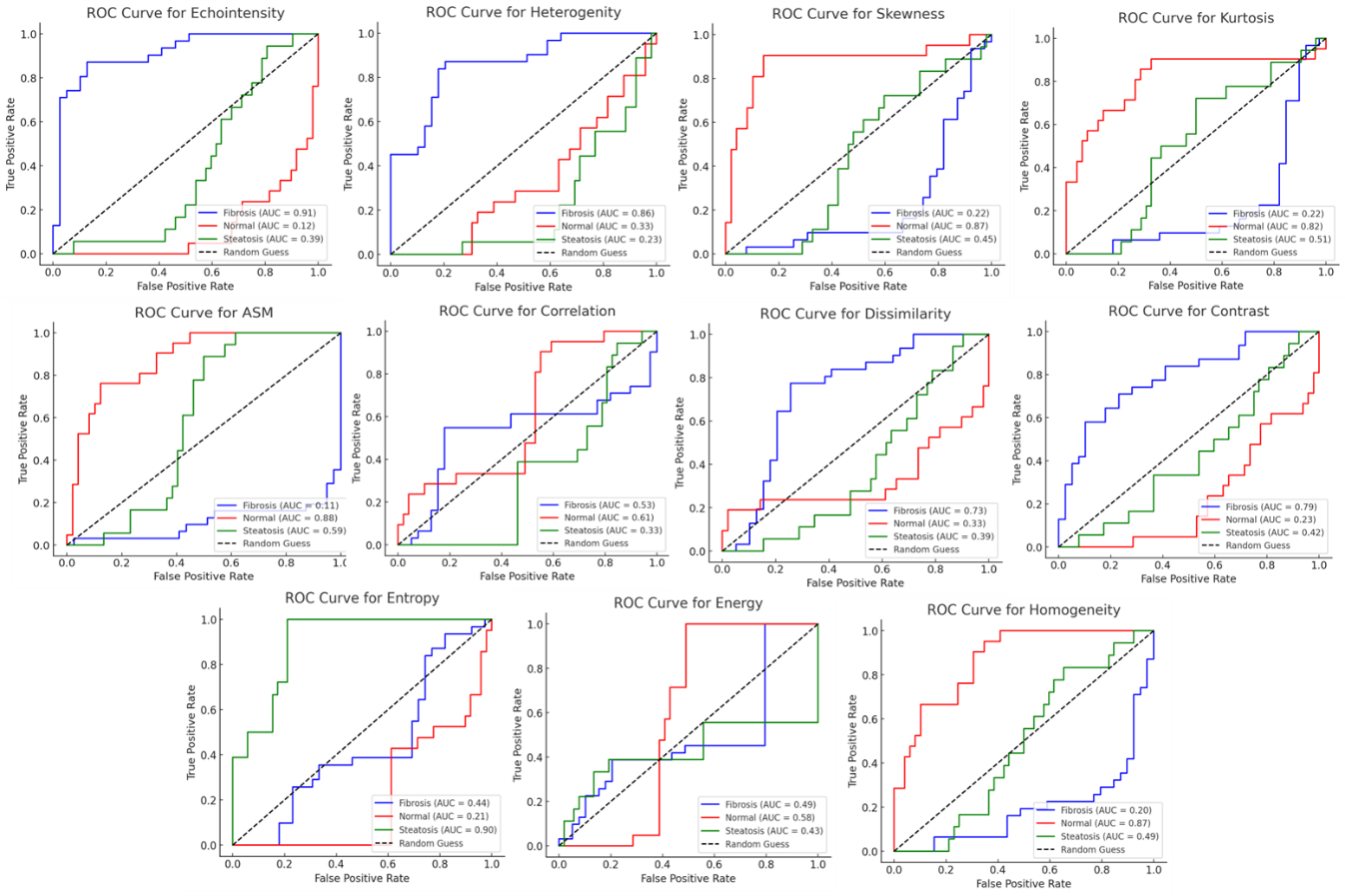


**Figure S1.** This figure presents receiver operating characteristic (ROC) curves comparing the diagnostic performance of various radiomic features in distinguishing between fibrosis, steatosis, and normal liver tissue. Each plot represents an individual radiomic feature, with the area under the curve (AUC) quantifying its classification accuracy. Higher AUC values indicate superior discriminatory power. Echo intensity (AUC = 0.91) and heterogeneity (AUC = 0.86) demonstrated strong differentiation for fibrosis, while homogeneity (AUC = 0.87) and skewness (AUC = 0.87) were more predictive of normal liver tissue. Features such as entropy and energy exhibited lower discriminatory performance. These results highlight the potential of radiomic analysis for non-invasive liver disease characterization using ultrasound imaging.

**
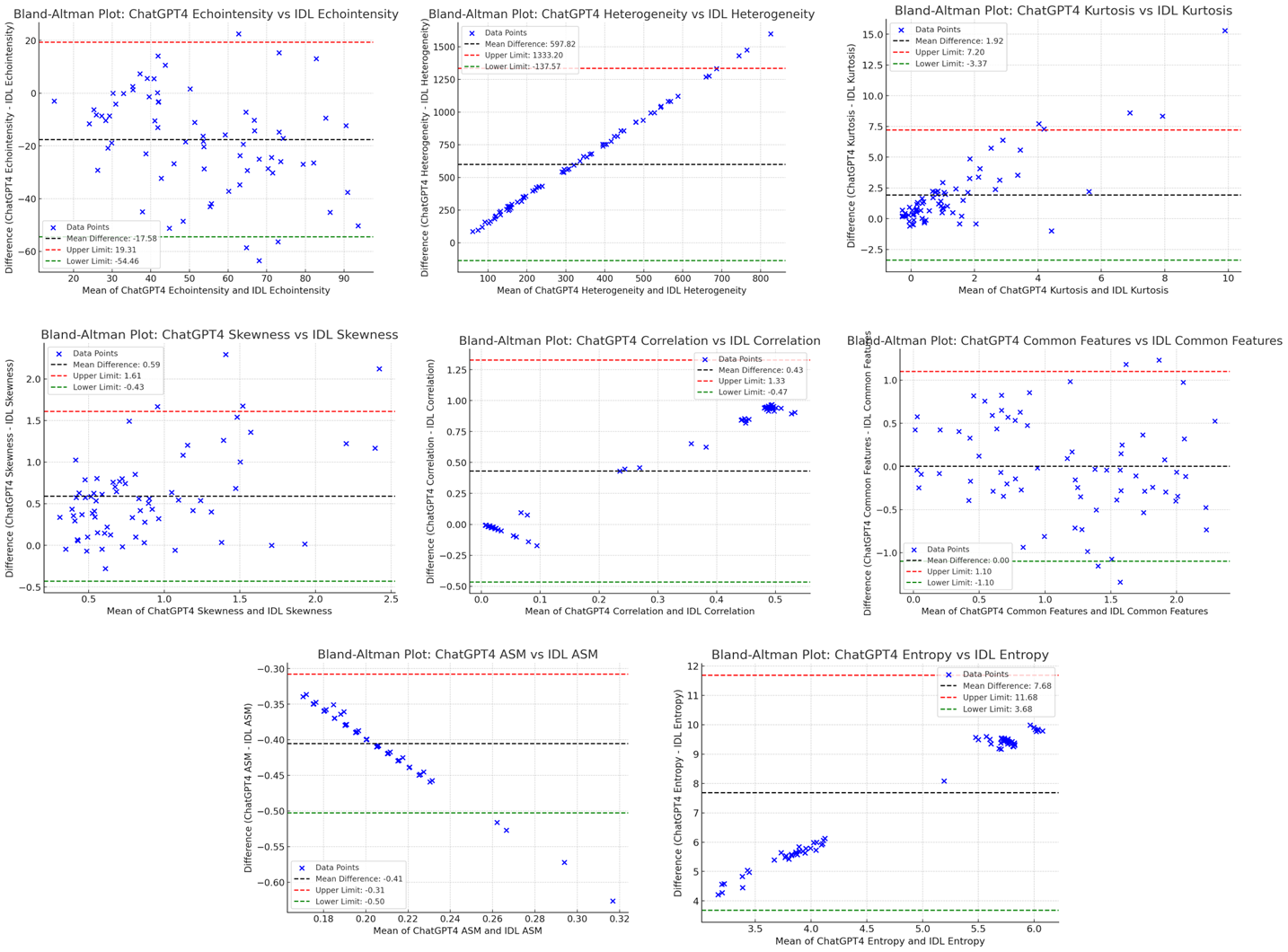
**

**Figure S2.** This figure presents Bland-Altman plots assessing the agreement between radiomic features extracted using ChatGPT-4 and IDL software. Each plot compares a specific radiomic feature, with the x-axis representing the mean values from both methods and the y-axis showing the difference between them. The solid black line denotes the mean difference (bias), while the dashed red and green lines indicate the upper and lower limits of agreement, respectively. Features such as heterogeneity and entropy exhibit strong agreement with minimal bias, while others, such as skewness and ASM, demonstrate greater variability. These results provide insights into the reliability and consistency of ChatGPT-4 in radiomic feature extraction for liver ultrasound analysis.
